# Chromatin- and Transcription-Related Factors Repress Transcription from within Coding Regions throughout the Saccharomyces cerevisiae Genome

**DOI:** 10.1371/journal.pbio.0060277

**Published:** 2008-11-11

**Authors:** Vanessa Cheung, Gordon Chua, Nizar N Batada, Christian R Landry, Stephen W Michnick, Timothy R Hughes, Fred Winston

**Affiliations:** 1 Department of Genetics, Harvard Medical School, Boston, Massachusetts, United States of America; 2 Banting and Best Department of Medical Research, University of Toronto, Toronto, Canada; 3 Département de Biochimie, and Centre Robert-Cedergren, Bio-Informatique Génomique, Université de Montréal, Montréal, Canada; Howard Hughes Medical Institute, United States of America

## Abstract

Previous studies in Saccharomyces cerevisiae have demonstrated that cryptic promoters within coding regions activate transcription in particular mutants. We have performed a comprehensive analysis of cryptic transcription in order to identify factors that normally repress cryptic promoters, to determine the amount of cryptic transcription genome-wide, and to study the potential for expression of genetic information by cryptic transcription. Our results show that a large number of factors that control chromatin structure and transcription are required to repress cryptic transcription from at least 1,000 locations across the S. cerevisiae genome. Two results suggest that some cryptic transcripts are translated. First, as expected, many cryptic transcripts contain an ATG and an open reading frame of at least 100 codons. Second, several cryptic transcripts are translated into proteins. Furthermore, a subset of cryptic transcripts tested is transiently induced in wild-type cells following a nutritional shift, suggesting a possible physiological role in response to a change in growth conditions. Taken together, our results demonstrate that, during normal growth, the global integrity of gene expression is maintained by a wide range of factors and suggest that, under altered genetic or physiological conditions, the expression of alternative genetic information may occur.

## Author Summary

Recent studies have shown that much more of the eukaryotic genome is transcribed into RNA than previously thought. In Saccharomyces cerevisiae, when particular factors are defective, cryptic promoters within several coding regions become active and produce shorter transcripts corresponding to the 3′ portions of genes. (Transcription proceeds from the 5′ end of genes to the 3′ end.) A comprehensive analysis of cryptic transcription identified the factors that normally repress this event. We find that at least 50 factors, many involved in chromatin structure and transcription, are required to repress cryptic transcription. Other results suggest that the potential for cryptic transcription is widespread, initiating from at least 1,000 locations across the S. cerevisiae genome. In mutants in which cryptic transcripts are produced, some of the transcripts are translated into proteins not normally made in unmodified, wild-type cells. Finally, in wild-type cells, a subset of cryptic transcripts is transiently induced following a nutritional shift, suggesting a possible role for cryptic transcription. Taken together, our results demonstrate that the normal pattern of gene expression is maintained by a wide range of factors and suggest that, under altered genetic or physiological conditions, the expression of alternative genetic information may occur.

## Abstract 3

Transcription from within open reading frames in*S. cerevisiae*, which is normally repressed by many chromatin- and transcription-related factors, has the potential to be widespread and to express alternative genetic information.

## Introduction

Several recent studies have demonstrated that transcription occurs across large eukaryotic genomes in a much more widespread and complex pattern than previously imagined. The recent findings of the ENCODE project, which analyzed transcription of 1% of the human genome [1], demonstrated the use of multiple transcription start sites and transcription across most sequences, including intergenic regions (reviewed in [2]). Many other recent studies have also identified extensive transcription across human sequences, including antisense transcription (reviewed in [3–5]). Similarly, in Drosophila melanogaster, recent studies estimate that 85% of the genome is transcribed, with extensive intergenic transcription and multiple transcription start sites [6]. Although the function of most of this pervasive transcription is currently not understood, there is evidence that a significant amount of it is regulated, raising the possibility that it is required for previously unknown modes of regulation or that it allows the expression of previously undetected genetic information [3–5]. Strong precedents exist for regulatory roles for intergenic transcription (for example, [7,8]; see [4,9] for recent reviews).

In Saccharomyces cerevisiae, similar to larger eukaryotes, several recent genome-wide studies have demonstrated widespread transcription across coding and noncoding regions [10–15]. In a small number of cases in S. cerevisiae, intergenic transcription [16–18], antisense transcription [19,20], and initiation within coding regions [21,22] have been shown to play biological roles. In addition to transcriptional events that occur in wild-type strains, other studies have revealed that transcription initiation can be activated from within coding regions in particular mutants [23,24]. Such initiation was originally observed in strains containing mutations in *SPT6* and *SPT16*, which encode conserved, essential transcription factors believed to be involved in nucleosome disassembly and assembly [23–27]. In an *spt6* mutant, the use of a transcription start site within the *FLO8* gene was shown to be dependent upon a consensus TATA element within the *FLO8* coding sequence, suggesting the existence of a cryptic promoter within *FLO8* that is normally repressed in a wild-type strain but becomes activated in an *spt6* mutant [23]. Evidence suggested that in *spt6* mutants, the failure to reassemble nucleosomes in the wake of elongating RNA polymerase II (RNAPII) allowed transcription initiation factors to bind to and activate cryptic promoters [23].

Several transcription factors are required to repress cryptic promoters in S. cerevisiae. An early study revealed that several different mutants allow cryptic initiation [23]. Subsequent analysis has suggested that the level of histone modifications in coding regions, as regulated by the Set2 histone methyltransferase and the Rpd3S histone deacetylase complex, also controls cryptic initiation [28–30] and that *set2Δ* mutations allow cryptic initiation in a large set of genes [31]. Additional work has identified other mutants that allow cryptic initiation, including *asf1* and *ctk1*, [32,33], as well as particular combinations of double mutants, revealing roles for other elongation factors, including the Paf1 complex, Bur1-Bur2, the HIR complex, Spt2, and Elf1 [34–36]. These studies suggest that the repression of cryptic promoters requires a variety of factors that play roles in transcription elongation and chromatin structure. These factors appear to be entirely distinct from those that suppress cryptic intergenic transcripts [37].

In this paper, we present the results of genome-wide approaches to comprehensively study cryptic transcription from within open reading frames (ORFs) in S. cerevisiae. First, we used both spontaneous mutant selection and a synthetic genetic array (SGA) screen to identify new mutations that allow cryptic transcription. These mutations have varying effects on the expression of a set of cryptic transcripts, suggesting the existence of different classes of cryptic promoters and mechanisms for their activation. Second, we used microarray analysis to identify cryptic transcripts throughout the S. cerevisiae genome that are activated in *spt6* and *spt16* mutants. These experiments showed that cryptic transcription is widespread, occurring in at least 1,000 genes (17% of all genes). We have also investigated the possibility of a physiological role for cryptic transcription, as it is not understood whether it represents unwanted transcription from fortuitous promoters that are activated only in mutants in which chromatin structure has been altered, or whether it serves a biological role in some cases, possibly to express different gene products. Here, we demonstrate that a number of cryptic transcripts expressed in an *spt6* mutant are translated into corresponding short proteins. In addition, we show that some cryptic transcripts are modestly activated in wild-type (*SPT6^+^*) strains upon a nutritional shift and that this activation is dependent upon Ras2. Taken together, our results show that cryptic transcription from ORFs can occur in a widespread fashion throughout the S. cerevisiae genome and suggest that some cryptic promoters may normally serve to express alternative genetic information during environmental changes.

## Results

### Comprehensive Identification of Mutants Permissive for Cryptic Transcription

**Figure 1:**
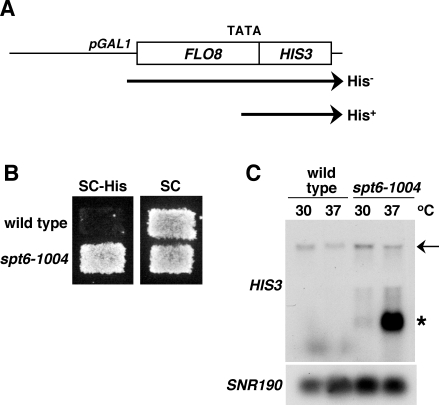
Detection of Cryptic Initiation with the *FLO8-HIS3* Reporter (A) Diagram of *FLO8-HIS3* reporter. The *FLO8* promoter is replaced by the *GAL1* promoter, and the 3′ end of the *FLO8* ORF is replaced with the *HIS3* ORF. The approximate position of the *FLO8* internal cryptic TATA site (base pair position +1,626) is shown. The expected *FLO8-HIS3* full-length and *HIS3* short transcripts are indicated beneath the diagram. (B) His^+^ phenotypes of wild-type and *spt6-1004* strains carrying the *FLO8-HIS3* reporter. Cells were replica-plated onto the indicated medium (SC or SC-His), and plates were grown at 30 °C for 5 d. (C) Northern analysis of wild-type and *spt6-1004* strains carrying the *FLO8-HIS3* reporter. RNA was isolated from cells either grown at 30 °C or shifted to 37 °C for 80 min. The probe for the northern analysis was generated against *HIS3*, and *SNR190* was used as a loading control. The arrow indicates full-length *FLO8-HIS3* RNA transcripts, and the asterisk indicates *HIS3* short transcripts resulting from cryptic initiation.

Previous results have shown that cryptic promoters are active in several mutants that impair transcription and chromatin structure. However, no systematic isolation of cryptic initiation mutants has been performed. To comprehensively identify factors that regulate cryptic promoters, we first constructed a reporter to allow easy detection of activation of the *FLO8* cryptic promoter. In this reporter, we replaced the region of *FLO8* 3′ of the cryptic transcription start site with the *HIS3* coding sequence (Figure 1A; Materials and Methods). The *HIS3* coding sequence was inserted out-of-frame with respect to the *FLO8* coding sequence, using the first ATG within *FLO8* that follows the cryptic start site. As this ATG is in the +2 reading frame, functional *HIS3* mRNA can only be made by transcription initiation at the *FLO8* cryptic start site (Figure 1A). In one version of this reporter, the normal *FLO8* promoter was replaced with the *GAL1* promoter to allow regulation of full-length *FLO8-HIS3* transcription by growth on different carbon sources and in a second version, the wild-type *FLO8* promoter was maintained. Both growth assays on plates lacking histidine and northern analysis demonstrated that the *FLO8-HIS3* fusion constitutes a sensitive reporter for mutants that allow cryptic initiation (Figure 1B and 1C).

**Figure 2:**
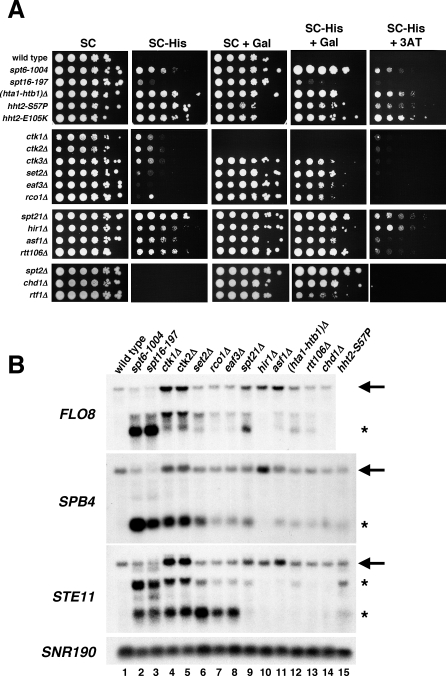
Analysis of Cryptic Initiation Mutants (A) His^+^ phenotypes of cryptic initiation mutants carrying the *FLO8-HIS3* reporter. Cells were spotted in a 10-fold dilution series from 1 × 10^8^ to 1 × 10^3^ cells/ml on the indicated medium. Growth on media containing galactose (Gal) induces expression of the full-length *FLO8-HIS3* construct, which can affect activation of the *FLO8* cryptic promoter in several mutants. Growth on media containing 3-aminotriazole (3AT), a competitive inhibitor of histidine, is indicative of higher expression levels of the *HIS3* transcript. Plates were grown at 30 °C for 6 d. The *ctk1* and *ctk2* mutants are unable to use galactose as a carbon source; therefore, they only grow on the plates with glucose as the carbon source regardless of the presence or absence of histidine in the growth medium. (B) Northern analysis of *FLO8*, *SPB4*, and *STE11* in cryptic initiation mutants. RNA was isolated from cells grown at 30 °C, except for the *spt6-1004* and *spt16-197* mutants, which were shifted to 37 °C for 80 min as indicated. *SNR190* was used as a loading control. Arrows indicate full-length RNA transcripts, and asterisks indicate short transcripts resulting from cryptic initiation.

Using *FLO8-HIS3*, we employed two methods to identify mutants that are permissive for cryptic initiation: direct selection and a screen of the S. cerevisiae nonessential deletion set (Materials and Methods). Direct selection was valuable for identification of strong mutations that are not in the deletion set, in particular, mutations in histone genes, described below. The deletion set screen allowed systematic testing of all nonessential genes. Overall, we identified mutations in 50 genes that allow cryptic initiation at *FLO8-HIS3* (Table 1). These 50 mutants are permissive for the *FLO8* cryptic promoter to varying degrees and several are dependent upon expression from the upstream *GAL1* promoter in the *FLO8-HIS3* reporter (Figure 2A). Overall, the majority of genes identified encode histones, regulators of histone gene expression, histone chaperones, and other factors implicated in transcriptional control.

**Table 1:**
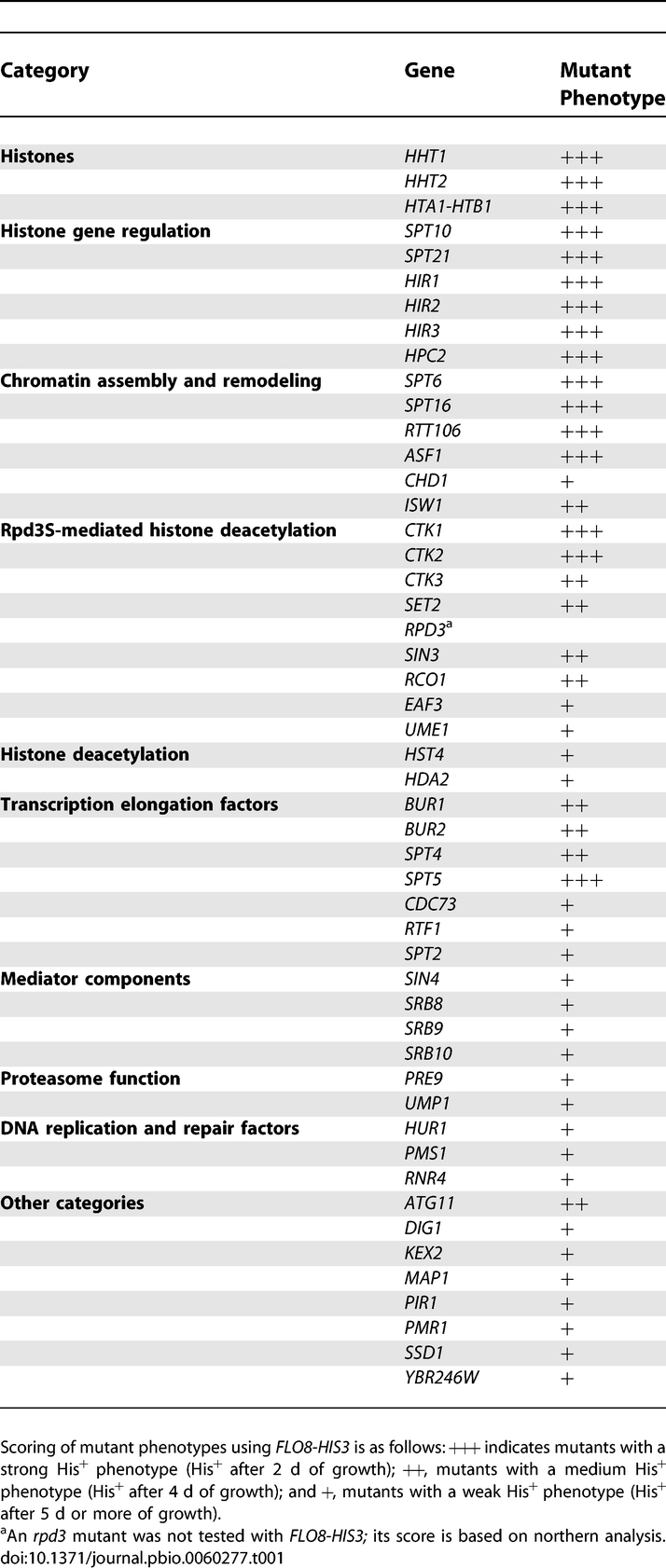
Genes Required for Repression of Cryptic Promoters

Among this large collection of mutants, histone H3 mutants are of particular interest as some identify previously unstudied changes in H3 that may play roles in transcription elongation. These H3 mutants are likely gain of function mutants, as deletion of either *HHT1* or *HHT2*, the genes encoding histone H3, causes only a very weak His^+^ phenotype with *FLO8-HIS3*, whereas the H3 mutants isolated by our selection are dominant and confer a strong His^+^ phenotype (Figure 2A, unpublished data). The majority of these H3 mutants are inviable when the second, wild-type H3 gene is deleted, suggesting that the H3 mutants are incapable of forming a functional nucleosome on their own (Table S1). One class of H3 mutants of interest includes four clustered changes in one region of H3: I51N, I51S, Q55H, and S57P. These changes are of interest due to their proximity to K56 of histone H3, whose acetylation has been shown to be important for resistance to DNA damaging agents, histone gene expression, and transcriptional silencing [38–42]. However, H3 K56 acetylation does not affect cryptic initiation, as an *rtt109*Δ mutation, which abolishes K56 acetylation [43–46], does not activate the *FLO8* cryptic promoter (unpublished data).

### Transcriptional Analysis Suggests Different Classes of Cryptic Promoters

To test whether the mutants we identified activate cryptic transcription from multiple genes, we performed northern analysis on 14 cryptic initiation mutants, examining transcription of *FLO8*, *SPB4*, and *STE11*, three genes previously shown to have cryptic promoters [23]. Our results show that there are different patterns of cryptic promoter activation among the mutants (Figure 2B). Most of the mutants express the *FLO8* short transcript, with the exceptions of *hir1Δ* and *chd1Δ* (Figure 2B, lanes 10 and 14; also see [35,36]), suggesting that in some cases, the *FLO8-HIS3* reporter is more sensitive in detecting cryptic initiation than northern analysis. Conversely, an *spt16-197* mutant appeared weakly His^+^ with the *FLO8-HIS3* reporter, whereas northern analysis indicated high levels of expression of the *FLO8* short transcript (Figure 2A and 2B, lane 3). This effect with *spt16-197* may be due to the slow growth of the *spt16-197* mutant. In addition, short transcripts could be detected for *SPB4* and *STE11* for most of the mutants, indicating that cryptic initiation was not specific to *FLO8*. However, there were differences in the pattern of cryptic transcription among the mutants tested. For example, *spt6-1004*, *eaf3Δ*, and *rtt106Δ* confer distinct patterns of activation of *FLO8*, *SPB4*, and *STE11* cryptic transcripts (Figure 2B, compare lanes 2, 8, and 13). These distinct patterns suggest that there are distinct classes of cryptic promoters and different mechanisms for their repression. Other evidence suggesting differential expression of cryptic transcripts has recently been described [47].

### Most Cryptic Transcription Mutants Have Normal Levels of Histone H3 K36 Methylation

**Figure 3:**
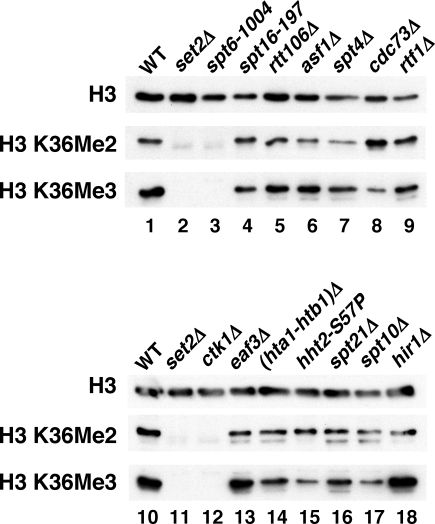
Western Analysis of Histone H3 K36 Methylation Levels in Cryptic Initiation Mutants Whole-cell extracts were prepared from cells grown at 30 °C, except for strains FY2425 and FY347, which were shifted to 37 °C for 80 min as indicated. Probes for the western analyses were generated with antibodies specific for total histone H3, dimethylated H3 K36, and trimethylated H3 K36. WT, wild type.

Recent results have shown that Set2-dependent methylation of histone H3 at K36 plays a role in the repression of cryptic transcription [28,47,29]. Furthermore, both H3 K36 dimethlyation and trimethylation have recently been shown to be defective in *spt6* and *spt16* mutants, as well as in *set2* mutants [28,48]. Therefore, we tested whether this histone H3 K36 methylation defect might be a common phenotype among cryptic transcription mutants. Our results show that, of 50 mutants tested, only five showed a significant decrease in total H3 K36 di- and trimethylation (*spt6-1004*, *set2Δ*, *ctk1Δ*, *ctk2Δ*, and *ctk3Δ*) (Figure 3, Table S2). The histone H3 K36 methylation defects in these five mutants have been previously reported [28,33,47–49]. We note that under our growth conditions, the *spt16-197* mutant had wild-type levels of H3 K36 di- and trimethylation, in contrast to a previous report [48], yet still showed a high level of cryptic transcription. These results show that the majority of the cryptic transcription mutants regulate at a step other than H3 K36 methylation.

### At least 1,000 Cryptic Transcripts are Produced in *spt6* and *spt16* Mutants

**Figure 4:**
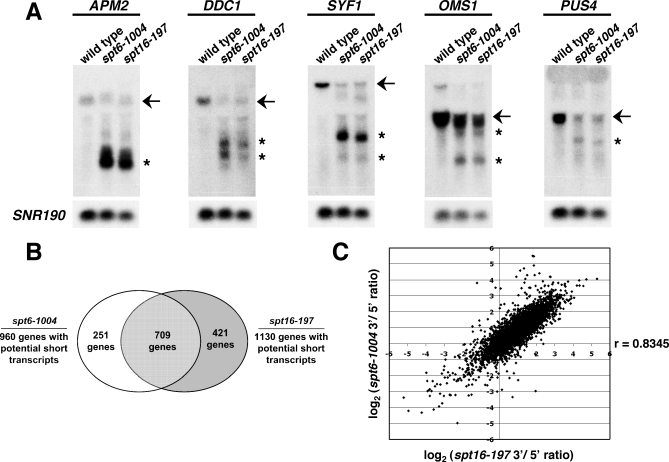
Microarray Analysis of Genes with Cryptic Promoters in *spt6* and *spt16* Mutants (A) Northern analysis of cryptic promoter genes identified by microarrays in *spt6-1004* and *spt16-197* mutants. RNA was isolated from cells after an 80-min temperature shift from 30 °C to 37 °C. Probes for northern analyses were generated with *APM2*, *DDC1*, *OMS1*, *PUS4*, and *SYF1*. *SNR190* was used as a loading control. Arrows indicate full-length RNA transcripts, and asterisks indicate short transcripts. (B) Venn diagram comparing the number of genes with cryptic promoters in *spt6-1004* and *spt16-197* mutants. Microarray results predict 960 genes to express short transcripts in an *spt6-1004* mutant and 1,130 genes to express short transcripts in an *spt16-197* mutant, based on a 3′/5′ ratio threshold of 2.5 for each gene. Among these, the overlap is 709 genes. (C) *spt16-197* versus *spt6-1004* plot of cryptic promoter microarray results. The log_2_ value of the 3′/5′ ratio for probe 6 of each gene in the *spt16-197* microarray was plotted against the log_2_ (3′/5′ ratio) value for probe 6 of the corresponding gene in the *spt6-1004* microarray. The correlation coefficient, *r*, between the two datasets is 0.8345.

Previous studies of cryptic initiation in an *spt6-1004* mutant identified only a few genes with cryptic promoters [23]. However, the frequency at which they were found among a small set of genes tested suggested that cryptic promoters may be widespread. To test this possibility, we assayed for cryptic transcription within ORFs on a genome-wide scale by microarray analysis. In these experiments, we compared mRNA from a wild-type strain to that from an *spt6-1004* mutant, using microarrays with six probes across each coding region (Materials and Methods). Using a stringent threshold (Materials and Methods), our results suggest that out of the 5,689 ORFs represented on the microarray, at least 960 genes (17%) have active cryptic transcription in the *spt6-1004* mutant (Figure S1; Table S3). As detailed in Materials and Methods, this method may unavoidably be biased towards identifying cryptic transcripts from genes with lower transcript levels, likely resulting in an underestimate of the actual number of cryptic transcripts (Materials and Methods; Figure S2). In support of the ability of the microarrays to identify genes with cryptic transcripts, we used northern analysis to test five genes predicted by the microarrays to have cryptic transcripts and found that all five indeed produce short transcripts (Figure 4A).

To test whether another mutant permissive for cryptic transcription allows production of the same large set of cryptic transcripts, microarray analysis was performed on the temperature-sensitive *spt16* mutant, *spt16-197*. These experiments identified approximately 1,130 genes predicted to have cryptic transcripts in the *spt16-197* mutant (Table S4). Between the *spt6-1004* and *spt16-197* results, there is a striking overlap (correlation coefficient *r* = 0.83, Figure 4B and 4C), indicating that these two mutants affect cryptic transcription similarly at most genes. Taken together, these results strongly suggest that approximately one sixth of all S. cerevisiae genes produce detectable cryptic transcripts in *spt6* and *spt16* mutants.

To determine whether the genes that produce cryptic transcripts share any particular traits, we examined several different characteristics of the genes that we identified in the *spt6* and *spt16* microarray experiments as having cryptic transcripts. With respect to the length of coding regions, the average length of the genes with cryptic transcription in both *spt6* and *spt16* mutants is 2.4 kb, significantly longer than the average length of the 5,869 genes on the microarray (1.5 kb; Wilcoxon rank-sum test, *p*-value < 2.2 × 10^−16^). The majority of genes with cryptic transcription also have lower transcriptional frequencies (for *spt6-1004*, average = 2.46 mRNA/hour [*p*-value < 2.2 × 10^−16^] and for *spt16-197*, 1.93 mRNA/hour [*p*-value , 2.2 × 10^−16^]) when compared with the whole genome (average = 7.57 mRNA/hour) [50]. The enrichment for longer genes with lower transcriptional frequencies was expected, as these two characteristics correlate, and our method for detection of cryptic transcripts enriched for genes with lower transcription levels.

In addition, we focused on TATA elements, both within coding regions and in 5′ noncoding regions. Since cryptic initiation within the *FLO8* coding region depends on the presence of a TATA element [23], we first tested whether genes showing cryptic transcription are enriched for those with TATA motifs in their coding sequence. We searched for the TATA consensus sequence in S. cerevisiae, TATA(A/T)(A/T)A(A/T)(A/G) [51]. We found that genes with at least one TATA element in their coding region are three times more likely to have a cryptic transcript in the *spt6-1004* mutant than genes without a TATA box (*p*-value < 2.2 × 10^−16^). We see an even stronger enrichment for the *spt16-197* mutant (*p*-value < 2.2 × 10^−16^) (Table S5). Given that our set of genes was enriched for those that are longer, we also examined whether these findings were still significant when corrected for gene size (longer genes are more likely to contain TATA motifs by chance) and found that they were (Figure S3). Thus, the genes with cryptic promoters identified by the *spt6-1004* and *spt16-197* microarray results suggest that cryptic transcription tends to be located in coding regions that contain TATA consensus sequences. We also classified the normal promoters of genes with cryptic promoters as to whether they have a TATA element or not. Genes with TATA elements tend to display more cell-to-cell and strain-to-strain variation in expression [52–57]. We found that cryptic transcripts are two times (Fisher exact test, *p*-value = 2 × 10^−12^) and 2.4 times (Fisher exact test, *p*-value = 2 × 10^−16^) more likely to be from genes with natural TATA-less promoters than from genes with TATA-containing promoters for the *spt6-1004* and *spt16-197* mutants, respectively, after correction for gene expression levels (Figure S4).

### Many Cryptic Transcripts Expressed in an *spt6* Mutant Are Translated

Given the large number of cryptic transcripts, it seemed likely that many of them would have the potential to encode proteins. We examined the potential for cryptic transcripts to be translated by mapping all ATGs in the three reading frames downstream of the 5′-most limit of transcription initiation established in the *spt6-1004* microarray analysis. For each of those ATGs, we mapped the first stop codons in the same frame to infer the peptide sequence that would result from translation from the internal ORFs. The results of this analysis (Table S6) show that most ORFs could encode proteins if the cryptic transcripts were to be translated: 820, 825, and 731 ORFs in frames +1, +2, and +3, respectively. However, the two alternative reading frames primarily encode short peptides, while, as expected, the +1 frame encodes much longer sequences.

**Figure 5:**
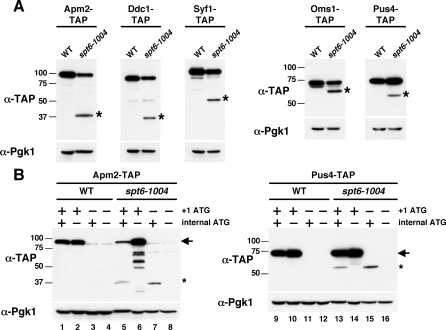
Analysis of Short Protein Expression in an *spt6* Mutant All Western analyses used whole-cell extracts prepared from cells after an 80-min temperature shift from 30 °C to 37 °C. Probes for the western analyses were generated with an antibody recognizing the TAP epitope tag. Pgk1 was used as a loading control. Arrows indicate full-length proteins, and asterisks indicate short proteins. WT. wild type. (A) Western analysis of short protein expression in an *spt6-1004* mutant. (B) Western analysis of short-protein expression in wild-type and *spt6-1004* strains containing *APM2* and *PUS4* ATG mutations. The lower molecular weight bands in lane 6 are likely to be degradation products.

To test directly whether genes with cryptic transcripts express proteins, we screened 146 genes that are predicted to have cryptic promoters and that have at least one internal ATG codon in the +1 frame located 3′ of the predicted cryptic start site. To screen these strains, we used the tandem affinity purification (TAP)-tagged set of S. cerevisiae strains in which each ORF is fused at its 3′ end to a sequence encoding the TAP epitope tag [58]. The TAP-tagged strains corresponding to the 146 selected genes were crossed to an *spt6-1004* strain to obtain TAP-tagged versions in both *SPT6^+^* and *spt6-1004* backgrounds. These strains were then screened by western analyses using an antibody recognizing the TAP tag to determine whether any altered proteins are made in the *spt6-1004* strains. We note that this method will only detect proteins produced by translation in the same reading frame as the full-length protein, because it requires that the TAP epitope tag be expressed. Our results show that 20 of the 146 genes tested produced a detectable shorter protein in the *spt6-1004* mutant but not in the *SPT6* ^+^ strain (Table S7, examples shown in Figure 5A). The short proteins were all in the size range predicted by the microarray results, and several of them encode domains with known activities lacking their normal amino-terminal sequences (Figure S5). Northern analysis of these genes verified that corresponding short transcripts of the appropriate sizes were indeed expressed in the *spt6-1004* mutant (Figure 4A; unpublished data).

To verify that the short proteins were produced by translation initiation from their corresponding short transcripts and were not simply degradation products of the full-length proteins, we analyzed the expression of short proteins made from two genes, *APM2* and *PUS4*. For each of these genes, we constructed and analyzed mutations that alter the initiation codon for both the normal, full-length protein and for the shorter protein and analyzed each by western analysis. Our results show that mutation of the normal ATG initiation codon eliminated expression of the full-length protein, but had no effect on expression of the short protein expression (Figure 5B, lanes 7 and 15). Furthermore, mutation of the internal ATG specifically abolished expression of the short protein (Figure 5B, lanes 6 and 14). We also observed that this mutation in *APM2* resulted in apparent degradation products (Figure 5B, lane 6), perhaps due to the amino acid change in the mutant protein. This mutation in *APM2* also causes increased expression of the full-length Apm2 protein specifically in the *spt6-1004* mutant and may be due to changes in either mRNA or protein stability. Taken together, these results demonstrate that at least a subset of transcripts expressed from cryptic promoters are translated to produce alternative, shorter proteins. The functions of these proteins are likely to be different from the full-length proteins because they often lose predicted protein domains (Table S8).

### A Subset of Cryptic Transcripts Are Expressed in Wild-Type Strains upon a Nutritional Shift

**Figure 6:**
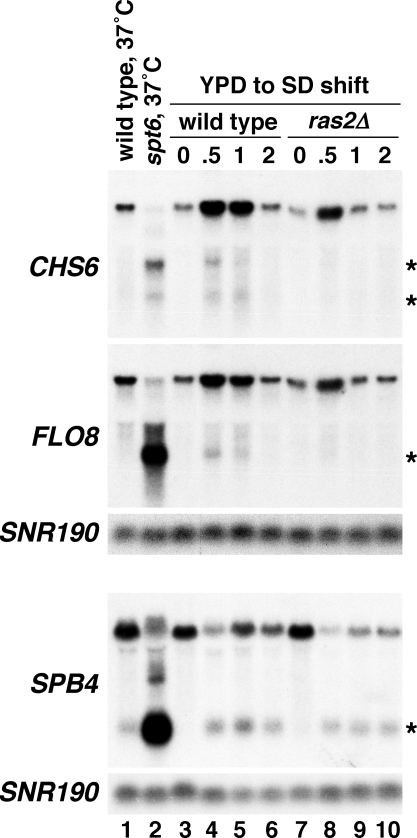
Northern Analysis of Cryptic Initiation during a Nutritional Shift from YPD Rich Medium to SD Minimal Medium for the Indicated Times Lanes 1 and 2 contain RNA isolated from cells shifted from 30 °C to 37 °C for 80 min as indicated. All other RNA was isolated from cells grown at 30 °C. *SNR190* was used as a loading control. Asterisks indicate short transcripts. *SNR190* served as a loading control.

The expression of the cryptic transcripts that we have identified is normally repressed in wild-type strains when cells are grown in rich medium. If some of the cryptic transcripts serve a biological function, however, they might be expressed in a wild-type background under particular growth conditions. To screen for such an effect, we used northern analysis to assay the transcription of 16 genes with cryptic transcripts under 20 different growth conditions. Most of these genes were selected from those shown to produce a protein from the cryptic transcript. The conditions tested included starvation for carbon, nitrogen, phosphate, or sulfate, as well as heat shock, high salt concentration, or exposure to different drugs such as 3AT or menadione. Of the 20 different growth conditions tested, one of them, a shift from rich medium (YPD) to minimal medium (SD), caused modest expression of cryptic transcripts in three of the 16 genes tested, *CHS6*, *FLO8*, and *SPB4* (Figure 6, lanes 3–6). For these genes, cryptic transcripts were detectable by 30 min after the shift, and for two of the genes, *CHS6* and *FLO8*, it was transient, no longer detectable by 2 h after the shift. In all cases, the level of the short transcript was clearly less than observed in the *spt6-1004* mutant, indicating that an *spt6* mutant represents an extreme condition for cryptic initiation in the genome, relative to what may be seen in a wild-type strain under different growth conditions.

Previous studies have shown that a nutritional shift from rich to minimal media causes other transient effects with very similar kinetics to what we have observed. Among these effects is the induction of translation of the transcription factor Gcn4 [59–61], which occurs in a Ras2-dependent fashion [62]. We therefore tested whether either Gcn4 or Ras2 plays a role in the expression of cryptic transcripts that we observe by assaying *gcn4Δ* and *ras2Δ* mutants during a nutritional shift. Although *gcn4Δ* did not affect cryptic transcript levels (unpublished data), our results showed that the expression of the *CHS6* and *FLO8* cryptic transcripts upon the nutritional shift was strongly Ras2-dependent, whereas the expression of the *SPB4* cryptic transcript appeared to be largely Ras2 independent (Figure 6, lanes 7–10). These results also suggest that the cryptic initiation induced at *CHS6* and *FLO8* after the nutritional shift is not simply the result of the increased expression of the full-length transcript seen for both genes following the media shift. Even though full-length expression of *CHS6* and *FLO8* is still greatly increased following the shift in the *ras2Δ* mutant, cryptic transcripts are not expressed, indicating some form of regulation of the cryptic promoters under these conditions. Thus, our results suggest that a subset of cryptic promoters can be specifically activated upon a nutritional shift in a Ras2-dependent fashion.

## Discussion

In this work, we have investigated cryptic transcription and its consequences in S. cerevisiae on a genome-wide scale. Our results have established that a large number of chromatin- and transcription-related factors are required to repress widespread cryptic transcription from within coding regions throughout the S. cerevisiae genome. Most of the cryptic transcripts contain ORFs, and our results suggest that when these cryptic transcripts are expressed, such as in an *spt6* mutant, many of them are translated to produce proteins that are not normally made. Thus, loss of Spt6 causes a dramatic change in the mRNAs and proteins produced genome-wide. Furthermore, a small subset of cryptic transcripts have been shown to be modestly expressed in wild-type strains during a nutritional shift. Taken together, these results demonstrate the widespread existence of cryptic transcription and the expression of alternative genetic information in S. cerevisiae.

Several results strongly suggest that multiple mechanisms control the expression of cryptic transcripts. Below, we discuss these possible mechanisms in terms of distinct classes of cryptic promoters. We note that our microarray results have established widespread cryptic transcription, but have not demonstrated that these transcripts all arise from cryptic promoters. However, based on our earlier studies of the *FLO8* and *SPB4* genes ([23] and unpublished data), we think it is likely that most or all of the cryptic transcripts identified are the result of activation of cryptic promoters. Testing this possibility will be the focus of future investigations. First, the mutants identified in this study vary greatly in their strength of cryptic initiation, based both on the *FLO8-HIS3* reporter and on northern analysis. Second, one of the most permissive mutants for cryptic initiation, *spt6-1004*, is known to impair at least two features of normal transcription elongation that individually contribute to repression of cryptic promoters: histone H3 K36 methylation [28,47,48] and the recruitment of the transcription factor Spt2 [35]. Consistent with this observation, both *set2Δ*, which abolishes histone H3 K36 methylation, and *spt2Δ* are less permissive for cryptic initiation than is *spt6-1004* (our results and [28,31,34,35]. In addition to these effects, *spt6-1004* likely causes other effects on chromatin structure [23,26]. Third, our results also showed that most mutations that allow cryptic initiation do not impair H3 K36 di- or trimethylation; therefore, loss of this histone modification is not the sole mechanism by which cryptic promoters are derepressed. This conclusion is consistent with recent studies that showed enhanced cryptic initiation in double mutants that lack Set2 and another factor, indicating that mechanisms other than histone H3 K36 methylation play an important role in this regulation [34]. Fourth, previous analysis has identified cases in which mutations that impair distinct aspects of transcription can combine to cause strong effects on cryptic initiation [34–36]. Finally, assay of a small set of cryptic promoters showed that they were activated in distinct patterns among different cryptic initiation mutants. For example, the pattern of cryptic initiation in mutants that impair Rpd3-mediated histone deacetylation was different from cryptic initiation in mutants affecting histone assembly (Figure 2B). Thus, cryptic promoters may be similar to normal promoters in terms of the complexity of regulation by distinct sets of factors, raising the possibility that additional transcription factors may regulate specific subsets of cryptic promoters. Consistent with this idea, our analysis of the *FLO8* cryptic promoter has shown that it requires a UAS-like element as well as a TATA element (V. Cheung and F. Winston, unpublished data).

The microarray experiments that we have performed suggest that there are at least 1,000 cryptic promoters in the S. cerevisiae genome that are activated in *spt6* or *spt16* mutants. The similarity between these two mutants suggests that they serve similar roles in normally repressing cryptic initiation, likely by helping to establish or maintain a repressive chromatin structure across coding regions [23,25]. Another recent set of microarray studies examined cryptic initiation in *set2Δ* mutants [31] and identified 621 genes with cryptic transcription on the sense strand. That study also identified 494 antisense transcripts, something not measured in our analysis. Similar to our results, the genes identified by Li et al. [31] were enriched for long genes transcribed at low level. Although we would expect that the cryptic promoters activated in *set2Δ* mutants would be a subset of those found in *spt6* and *spt16*, only 45% of those found in *set2Δ* were found in *spt6*. This degree of overlap, while still quite significant, was likely affected, at least in part, by differences in the microarrays and analysis of the datasets. The smaller number of cryptic promoters in *set2Δ* mutants compared to *spt6* and *spt16* fits with our results that mechanisms beyond histone H3 K36 methylation control cryptic initiation. The possible role of antisense transcripts is unknown, although recent studies have demonstrated roles in transcriptional regulation [19,20].

Other evidence suggests that promoters within coding regions occur on a wider scale than indicated by our microarrays of *spt6* and *spt16* mutants. One study, that examined the S. cerevisiae transcriptome in a wild-type strain by serial analysis of gene expression (SAGE), identified 384 genes with transcription start sites located within the 3′ half of the coding region [12]. Only 55 of these 384 genes (14.3%) were identified in our *spt6-1004* microarrays to express short transcripts. This small overlap is expected, as our experiments were designed to identify cryptic promoters activated specifically in *spt6* mutants. In addition, our *spt6-1004* microarrays were designed to detect short transcripts only from the sense strand, while the SAGE analysis was able to detect both sense and antisense short transcripts. More thorough microarray and transcriptome analysis of additional cryptic initiation mutants and other growth conditions will provide a more comprehensive map of cryptic promoters in the yeast genome.

The question still remains as to why so many cryptic promoters are found in the S. cerevisiae genome and what role they serve, if any. We can envision at least four possible roles for cryptic promoters, none of which are mutually exclusive, as all are possible for different subsets. First, some cryptic promoters may direct the expression of gene products that carry out specific functions, being expressed in response to particular environmental changes. In this way, use of cryptic promoters would be analogous to other mechanisms of expressing different genetic information, such as alternative splicing or use of internal ribosome entry sites. Although our results have not demonstrated a function for a product of cryptic initiation, precedent exists for using an internal promoter to express an alternative protein, sometimes under particular growth conditions [22,63–66]. In mammalian cells, the use of alternative promoters has been shown to have numerous roles in normal gene expression and in disease-associated genes [67]. Other results have also shown the potential to express shorter gene products in response to an environmental change [68]. Our results, showing that many cryptic transcripts are translated and that some cryptic promoters are activated by a nutritional shift, also fit with this possibility. We note that we did test for evidence of conservation between S. cerevisiae genes with cryptic transcription and S. bayanus orthologs, but did not detect any significant reduction in either synonymous or nonsynonymous changes in genes with cryptic transcription when compared to genes without cryptic transcription, but of similar length (unpublished data). Second, the information expressed from cryptic promoters may provide the potential for an adaptive mechanism in which, under appropriate selective conditions, expression of such products would enable improved growth or survival, thereby facilitating evolutionary genetic changes. Such an idea was previously suggested for the yeast prion [*PSI*+], which affects the fidelity of translational termination and thus allows for the possible production of novel protein products [69–71]. Strains containing [*PSI*+] can acquire complex phenotypic traits distinct from [*psi*−] strains, and when outcrossed to wild-type strains, these phenotypic traits can sometimes be maintained even after treatment to remove [*PSI*+] [70,71]. A possible role for intergenic RNAs has also been previously suggested [72]. Third, some cryptic promoters may serve to regulate transcription or control chromatin structure without producing a functional gene product. A previous study demonstrated that a promoter within *PRY3* of S. cerevisiae serves to repress *PRY3* expression during mating [21]. In this case, transcription from the internal promoter does not appear to play any functional role. In other cases, the act of transcription may alter chromatin structure in some beneficial way, as previously suggested [68]. Finally, some cryptic promoters may be “noise,” existing as one of many transcriptional events that serve no apparent biological role [73]. In such a scenario, a significant role of the genes we identified in our screen would be to minimize such “noise,” similar to that of Trf4, Air1, Air2, and components of the exosome in the removal of cryptic intergenic transcripts [37]. Given the very large number of cryptic promoters in S. cerevisiae, it seems reasonable to speculate that all of these reasons and others may turn out to be true. The analysis of specific cryptic promoters will likely yield additional insights into their roles and into previously unknown aspects of gene expression.

## Materials and Methods

### S. cerevisiae strains and media.

All S. cerevisiae strains are listed in Table S9. Strains with the prefix “FY” are isogenic with a *GAL2* ^+^ derivative of S288C [74]. Strains were constructed by standard methods, either by crosses or by transformations [75]. The *spt6-1004* [23]), *spt16-197* [76], *(hta1-htb1)Δ0::LEU2* [77], *spt2Δ0::kanMX* [35], *spt21Δ0::kanMX* [78], *spt4Δ0::URA3* [79], and *RAS2^val19^* [80] alleles have been previously described. The *can1Δ0::STE2pr-LEU2* allele was generously provided by the Boone lab [81]. The *ctk3Δ0::kanMX*, *set2Δ0::kanMX*, *eaf3Δ0::kanMX*, *rco1Δ0::kanMX*, *asf1Δ0::kanMX*, *rtt106Δ0::kanMX*, *chd1Δ0::kanMX, cdc73Δ0::kanMX*, *rtf1Δ0::kanMX, ras2Δ0::kanMX*, and *ygr117cΔ0::kanMX* deletion mutations are from the S. cerevisiae haploid nonessential genome deletion library [82], and the deletions were verified to be correct by PCR. The *ctk1Δ0::URA3*, *ctk2Δ0::URA3*, *chd1Δ0::HIS3*, *hir1Δ0::LEU2*, and *hir1Δ201::kanMX* deletion mutations were constructed by replacing the ORFs with *URA3*, *HIS3*, *LEU2*, or *kanMX* by standard methods [83–85]. The *spt10-302::URA3* allele consists of a Tn10-LUK transposon [86] inserted at the *SPT10* locus. The *[SPT10::URA3]dup* duplication allele was constructed by integrating plasmid pFW216 (a derivative of pRS306 containing *SPT10* and *URA3*) at the *ura3-52* genomic locus [87,88]. To construct the *hht2-S57P* allele, a 1.9-kb SmaI-EcoRI fragment containing *HHT2* and *HHF2* from plasmid pDM18 was ligated into vector pRS306 (*URA3*) [88,89]. The TCT codon (Ser) at base pair position +172 of *HHT2* (relative to the +1 ATG start codon) was mutated to a CCT codon (Pro) by site-directed mutagenesis (QuikChange kit, Stratagene). The resulting plasmid was used to replace the wild-type *HHT2* allele in strain FY2716 by two-step gene replacement. The presence of the mutant *hht2-S57P* allele in the genome was verified by sequencing. Strain FY2724 contains a point mutation in *HHT2* changing the GAA codon (Glu) at base pair +316 to an AAA codon (Lys) and was created by UV mutagenesis of strain FY2713 and verified by sequencing.

To construct the *kanMX-GAL1pr-flo8-HIS3* reporter, a 2-kb cassette containing the *kanMX* marker and the *GAL1* promoter was amplified by PCR from plasmid pFA6a-*kanMX6-PGAL1* [90]. This cassette was used to transform strain FY2425 by integration at the *FLO8* promoter, replacing base pairs −1,147 to −1 (relative to the *FLO8* +1 ATG start codon), to create strain FY2174. The *HIS3* ORF (663 bp) was amplified by PCR from plasmid pRS403 [84] and transformed into strain FY2174 at the genomic *FLO8* locus, replacing the 3′ end of the *FLO8* ORF and the first 105 bp of the 3′ UTR (base pairs +1,727 to +2,505 of *FLO8* relative to the +1 ATG start codon). Successful transformants were selected on SC-His medium and verified by PCR. The *HIS3* ORF is inserted out-of-frame with respect to the *FLO8* ORF and is inserted 3′ of both the internal *FLO8* TATA element (+1,626 to +1,631) and the cryptic transcription initiation sites of the *FLO8* short transcript (+1,679 to +1,685) [23]. To construct the *flo8-HIS3* reporter, the *HIS3* ORF was transformed into strain FY2425 and inserted at the *FLO8* genomic locus as described above.

The *APM2-TAP::His3MX*, *DDC1-TAP::His3MX*, *OMS1-TAP::His3MX*, *PUS4-TAP::His3MX*, and *SYF1-TAP::His3MX* alleles are from the S. cerevisiae TAP-tagged library [58]. The *apm2–1-TAP::His3MX* allele contains a point mutation in the in-frame ATG codon at base pair position +1,420 of *APM2* (relative to the +1 ATG start codon), changing it to a TTG codon (Leu). The *apm2–2-TAP::His3MX* allele contains three point mutations at the +1 ATG start codon of *APM2*, changing it to a CGT codon. The *apm2–3-TAP::His3MX* allele contains both the +1 ATG and the +1,420 ATG mutations in *APM2*. The *pus4-1-TAP::His3MX* allele contains a point mutation in the in-frame ATG codon at base pair +478 of *PUS4* (relative to the +1 ATG start codon), changing it to a GTG codon (Val). The *pus4–2-TAP::His3MX* allele contains three point mutations at the +1 ATG start codon of *PUS4*, changing it to a CGT codon. The *pus4-3-TAP::His3MX* allele contains both the +1 ATG and the +478 ATG mutations in *PUS4*. All ATG mutations were constructed by a two-step gene replacement using a previously described method [91] and verified by sequencing.

For liquid cultures, strains were grown in either YPD rich medium (1% yeast extract, 2% peptone, and 2% glucose) or SD minimal medium (0.15% yeast nitrogen base, 0.5% ammonium sulfate, and 2% glucose) as indicated. Synthetic complete media plates (SC) and synthetic complete drop-out media plates (SC-His) were made as previously described [75]. SC + Gal plates and SC-His + Gal plates were made using 2% galactose instead of glucose as the carbon source. For the spontaneous mutant selection, 3-aminotriazole (3AT) was added to SC-His plates at the concentrations described below.

### Isolation of cryptic initiation mutants.

Cryptic initiation mutants were isolated using the following three methods: spontaneous mutant selection, synthetic genetic array (SGA) analysis with the S. cerevisiae genome nonessential deletion set [82,92], and direct testing of candidate genes. Spontaneous mutant selection was performed using the parental wild-type strains FY2393, FY2713, FY2717, and FY2718, each containing the *kanMX-GAL1pr-flo8-HIS3* reporter. Parental strains were grown overnight in 5-ml YPD cultures at 30 °C, washed twice in water, and then either 1 × 10^7^ cells or 1 × 10^8^ cells from each culture were plated on SC-His media plates containing 0, 1, 2, 3, 4, 5, or 10 mM 3AT. Plates were either UV-irradiated (5,000 μJ/cm^2^) or left untreated, and then grown at 30 °C to select for His^+^ mutants. Potential His^+^ cryptic initiation mutants were single-colony purified and retested to verify their His phenotype. Mutant genes were identified by diploid complementation, plasmid complementation, linkage analysis, and cloning by plasmid complementation with an S. cerevisiae genomic library [93]. A total of 254 different mutants were isolated, and 226 of them were identified as belonging to the following groups: *SPT21*, *SPT10*, *HTA1-HTB1*, *HHT1*, *HHT2*, *HIR1*, *HIR2*, *HIR3*, *HPC2,* and mutations linked to the *kanMX-GAL1pr-flo8-HIS3* reporter. SGA analysis was performed as previously described [92], using the query strain L1102 and screening for deletion mutants that allowed growth on SC-His media plates. Potential positive candidates from SGA analysis were individually crossed with strain FY2506, and their His phenotype was verified by tetrad analysis.

### Microarray design, hybridization, and analysis.

Probe sequences corresponding to 5,869 ORFs of the S. cerevisiae genome were submitted to Agilent Technologies for microarray production. Each ORF was represented by six 60-mer probes spaced evenly along its coding sequence, with the most-5′ probe beginning at base pair position +1 (relative to the +1 ATG start codon) and the most-3′ probe ending at the final stop codon. Strains FY80 and FY2425 were used for four independent *spt6* microarray experiments, and strains FY70 and FY347 were used for two independent *spt16* microarray experiments. Experimental pairs were performed in dye reversal. Wild-type and mutant cells were grown in YPD medium at 30 °C to mid-log phase (1–3 × 10^7^ cells/ml), shifted to 37 °C for 80 min, and then harvested as previously described [94] Sample preparation, labeling, and hybridization of microarrays were performed as previously described [94,95]. Microarray images were acquired and spots quantified with a GenePix 4000B microarray scanner and 3.0 software, respectively (MDS Sciex). Spatial detrending and variance stabilization normalization of raw microarray data were performed as previously described [95]. Genes were detected as expressing short transcripts in either the *spt6* or the *spt16* mutant using the following criteria. The mutant/wild-type ratio was calculated for each probe on the microarray using the normalized spot fluorescent intensity values. For each ORF, the 3′/5′ ratio was calculated by dividing the mutant/wild-type ratio of the most-3′ probe by the mutant/wild-type ratio of the most-5′ probe. Genes with high 3′/5′ ratios were predicted to express short transcripts, whereas genes with low 3′/5′ ratios (close to 1.0) were predicted to not express short transcripts. The location of the internal transcription start site for genes generating short transcripts was estimated by calculating the mutant/wild-type ratio of each probe in the gene relative to the corresponding ratio of the most-5′ probe. Based on the microarray results for five genes previously known to express short transcripts in an *spt6* mutant (*FLO8*, *SPB4*, *STE11*, *RAD18*, and *VPS72*) [23], a 3′/5′ ratio threshold was set at 2.5, where only genes with a 3′/5′ ratio greater than 2.5 in either all four *spt6* microarray experiments or both *spt16* microarray experiments were predicted to express a short transcript. Using this criterion, 960 genes were predicted to express short transcripts in an *spt6* mutant, and 1,130 genes were predicted to express short transcripts in a *spt16* mutant. It is likely, though, that even more cryptic promoters exist, as the method of calculation likely and unavoidably discriminates against the identification of cryptic transcripts from highly transcribed genes. This discrimination arises from the fact that the hybridization signal from the 3′ probe is the sum of the signals for both the full-length and cryptic transcripts. Thus, for genes with a high level of the full-length transcript, the level of a cryptic transcript would need to also be high to be detectable. The 3′/5′ ratio from the microarray results are shown plotted according to expression levels in Figure S2. In support of a greater number of cryptic transcripts, when a more relaxed threshold was used (3′/5′ ratio of 2.0 rather than 2.5), 620 additional genes were predicted to express cryptic transcripts. When five genes were randomly selected from these 620 genes, four of them expressed short transcripts as detected by northern analysis (unpublished data). However, it is clear that not all genes produce cryptic transcripts, as northern analysis of ten other genes at random showed that only one produced a detectable cryptic transcript (I. Ivanovska, J. Pamment, and F. Winston, unpublished data).

### Northern analysis.

mRNA preparation and northern hybridization analysis were performed as previously described [96]. Unless otherwise indicated, RNA was prepared from cells grown in YPD at 30 °C to mid-log phase (1–3 × 10^7^ cells/ml). For temperature shift experiments, cells were grown in YPD to mid-log phase at 30 °C and then shifted to 37 °C for 80 min. For media shift experiments, cells were grown in YPD to mid-log phase at 30 °C, washed twice with SD, and then grown in an equivalent volume of SD at 30 °C for the indicated times. Double-stranded northern probes were amplified by PCR from genomic DNA and were designed to hybridize to the 3′ ends of *FLO8* (+1,515 to +2,326), *SPB4* (+1,605 to +1,812), *STE11* (+1,868 to +2,110), *APM2* (+1,449 to +1,786), *DDC1* (+1,489 to +1,739), *OMS1* (+1,084 to +1,351), *PUS4* (+861 to +1,134), *SYF1* (+2,032 to +2,525), and *CHS6* (+1,917 to +2,295). A probe for *SNR190* (+1 to +190) was used as a loading control for all northern analyses. Because the probes are double stranded, they could anneal to either sense or antisense transcripts. The base pair positions given for each probe is relative to the +1 ATG start codon of the respective gene.

### Western analysis.

For Western analysis of histone H3 and H3 K36 methylation, whole-cell protein extracts were prepared as previously described [79]. The protein concentration of extracts was determined by Bradford assay (Bio-Rad). Fifty micrograms of whole-cell extracts were separated on 15% acrylamide SDS-PAGE gels, transferred to immobilon-P membrane (Millipore), and analyzed by immunoblotting as previously described [48]. Antibodies were used that recognized total histone H3 (1:5,000 dilution, Abcam), dimethylated H3 K36 (1:10,000 dilution, Upstate), and trimethylated H3 K36 (1:10,000 dilution, Abcam). Antibodies were detected by chemiluminescence (PerkinElmer).

For western analysis of TAP-tagged proteins, whole-cell protein extracts were prepared as follows: 50 ml of cells were grown in YPD at 30 °C to mid-log phase (1–3 × 10^7^ cells/ml) and then shifted to 37 °C for 80 min. Cells were washed twice with wash buffer (20 mM Tris-Hcl, 150 mM NaCl [pH 7.5]) and resuspended in 400 μl of lysis buffer (50 mM Hepes-KOH [pH 7.5], 150 mM NaCl, 10% glycerol, 0.5% NP-40, 1 mM EDTA, 1 mM PMSF, 2 μg/ml Leupeptin, 2 μg/ml Pepstatin A). One milliliter of glass beads was added, and cells were lysed by vortexing in an Eppendorf multihead shaker 5432 for 40 min at 4 °C. The cell lysate was spun out through a hole punctured in the bottom of the tube, by spinning for 2 min at 1,000 rpm. The lysate was spun for 5 min at 14,000 rpm, and the supernatant was saved and spun again for 15 min at 14,000 rpm. The supernatant was saved from this final spin and used for western analysis. Total protein concentration of extracts was determined by Bradford assay (Bio-Rad). Equal amounts of whole-cell extracts were separated on 8% acrylamide SDS-PAGE gels, transferred to immobilon-P membrane (Millipore), and analyzed by immunoblotting as previously described [79]. The TAP tag was detected by chemiluminescence (PerkinElmer Life Sciences) using the peroxidase anti-peroxidase antibody (1:5,000 dilution, Sigma). Pgk1 was used as a loading control and visualized with anti-Pgk1 antisera (Molecular Probes) that was generously provided by Angelika Amon's laboratory.

### Analysis of open reading frames.

To examine which protein domains are present and lost, we obtained data on proteins from SGD (ftp://genome-ftp.stanford.edu/pub/yeast/sequence_similarity/domains, last updated on September 25, 2007) and mapped them onto the proteins encoded by genes with cryptic transcription initiation. We considered the first ATG after the most-5′ limit of the cryptic transcript as a conservative limit for the length of the short protein being produced; i.e., cases in which a minimal number of residues would be lost. A domain was called to be absent if the position of the ATG was downstream of the domain start site. Using published data on protein domains that are at the physical interface of the interacting partners [97], we also examined whether these lost domains are known to mediate physical interaction among proteins. Finally, in order to estimate how common these domains are among yeast proteins, we tabulated how many proteins in the genome have these domains.

### Accession number.

The microarray data, accession number GSE12272, can be found at GEO (http://www.ncbi.nlm.nih.gov/geo/).

## Supporting Information

Figure S1 Clustergram Analysis of *spt6-1004* Transcription Clustergram showing normalized *spt6-1004*/wild-type ratios (as median-subtracted asinh values; [98]) for individual probes from the 960 genes we classified as having short transcripts in an *spt6-1004* mutant. The color scale shown spans from asinh −2 to +2. Numbers shown next to the color bar indicate corresponding ratios in the linear domain and are rounded to the nearest integer.
(49 KB PDF)

Figure S2 Possible Underestimation of Cryptic Transcripts Cryptic transcripts may be less likely to be detected for genes with abundant transcripts because the abundance of cryptic transcripts does not scale with that of the full transcript. The ratio of 3′ to 5′ of hybridization intensity is shown as a function of transcript abundance. The ratio was calculated as defined in the text. In grey are ratios of intensities for probe 6 and 5 and in black for probes 6 and 1. The red line indicates the cutoff for calling the presence of a clear cryptic transcript (2.5). (A) *spt6-1004* mutant; (B) *spt16-197* mutant.
(7.41 MB PDF)

Figure S3 TATA Motifs in ORFs and Cryptic Transcription The presence of a TATA motif in the coding sequence of a gene increases the probability of cryptic initiation of transcription in that gene, independent of the size of the gene. Genes were separated by size classes corresponding to each of the ten intervals corresponding to the ten quantiles of the size distribution. The expected number of genes was calculated as the product of the fraction of genes with a TATA motif in this size interval times the number of genes with cryptic transcripts in that interval. The observed number represents the fraction of genes with a TATA motif in the coding sequence that produce cryptic transcript. (A) *spt6-1004* mutant; (B) *spt16-197* mutant.
(466 KB PDF)

Figure S4 TATA Motifs in Promoters and Cryptic Transcription Genes with a TATA box in their promoter are less likely to produce cryptic transcripts, and the occurrence of a TATA box occurs independently from transcript abundance. Genes were separated by expression classes corresponding to each of the ten intervals corresponding to the ten quantiles of the distribution. The expected number of genes with a TATA promoter was calculated as the product of the fraction of genes with a TATA promoter for this expression level interval times the number genes with cryptic transcript in that interval. If the presence of a TATA promoter was independent from the production of a cryptic transcript, the fraction of genes that produce a cryptic transcript and that have a TATA promoter should be proportional to the fraction of genes with a TATA promoter in this size interval. (A) *spt6-1004* mutant; (B) *spt16-197* mutant.
(517 KB PDF)

Figure S5 Examples of Proteins Made in *spt6-1004* Mutants from Cryptic Transcripts Shown are proteins found to be expressed from cryptic promoters (Table S8). The gray boxes designate the portions that are in the shorter proteins. The orange line represents the full-length protein that is made from the wild-type transcript.
(172 KB PDF)

Table S1 Histone H3 Mutants That Allow Cryptic Initiation (22 KB DOC)

Table S2 Histone H3 Di- and Trimethylation Levels in Mutants (20 KB XLS)

Table S3 *spt6-1004* Microarray Results (5.62 MB XLS)

Table S4 *spt16-197* Microarray Results (5.62 MB XLS)

Table S5 TATA Consensus Sequences in Coding Regions (22 KB DOC)

Table S6 Translation of Coding Regions in Cryptic Transcripts (1.01 MB XLS)

Table S7 Genes Identified That Express Short Proteins in an *spt6-1004* Mutant (22 KB DOC)

Table S8 Domains Lost in Potential Translation Products in *spt6-1004* Mutants (1.34 MB XLS)

Table S9 S. cerevisiae Strains (33 KB DOC)

## Abbreviations

ORF: open reading frame
SGA: synthetic genetic array
TAP: tandem affinity purification
