# Supplementary material for: Chromatin- and Transcription-Related Factors Repress Transcription from within Coding Regions throughout the Saccharomyces cerevisiae Genome
Source: PLoS Biol. 2008 Nov 11;6(11):e277. doi: 10.1371/journal.pbio.0060277 (PMC2581627; doi:10.1371/journal.pbio.0060277)
Supplement: Supplementary file 2 [file pbio.0060277.sg002.pdf]

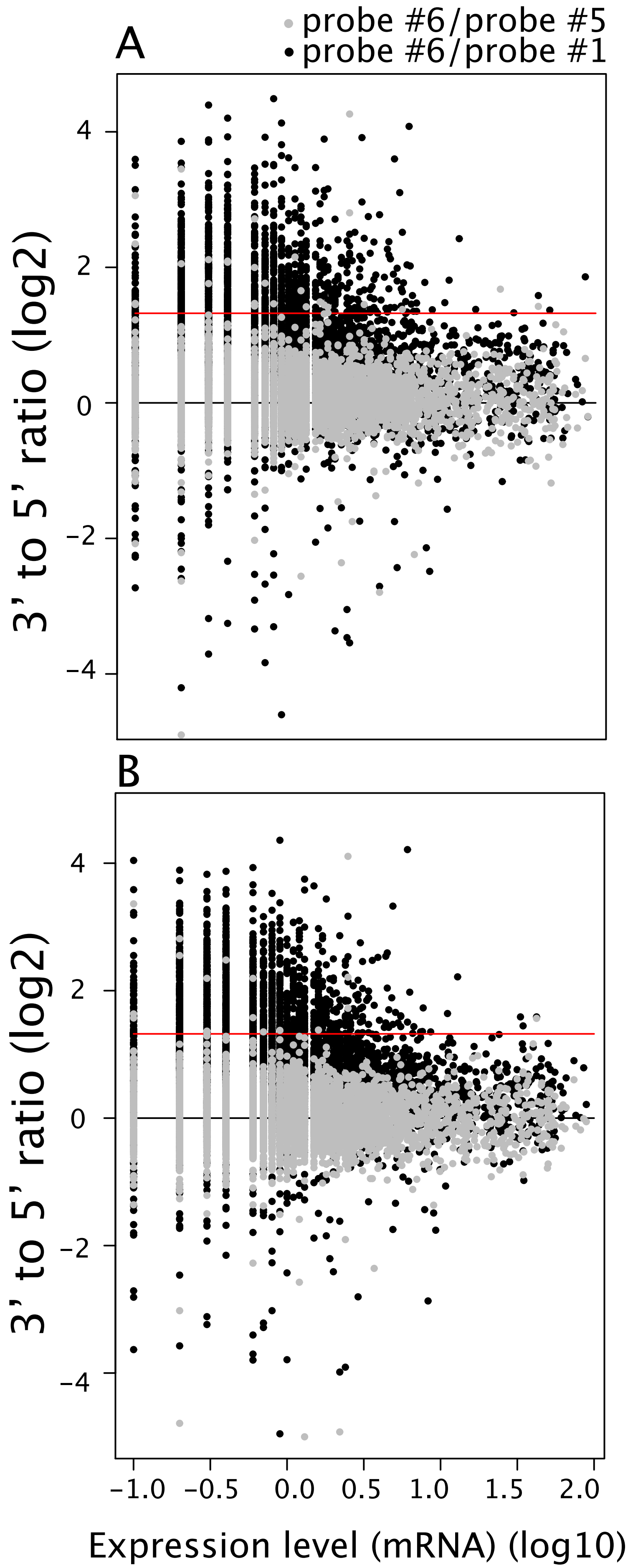
A
probe #6/probe #5
probe #6/probe #1
3' to 5' ratio (log2)
4
2
0
-2
-4
B
3' to 5' ratio (log2)
4
2
0
-2
-4
-1.0
-0.5
0.0
0.5
1.0
1.5
2.0
Expression level (mRNA) (log10)
