# Supplementary material for: Chromatin- and Transcription-Related Factors Repress Transcription from within Coding Regions throughout the Saccharomyces cerevisiae Genome
Source: PLoS Biol. 2008 Nov 11;6(11):e277. doi: 10.1371/journal.pbio.0060277 (PMC2581627; doi:10.1371/journal.pbio.0060277)

Fig. S3. Cheung et al. 2008

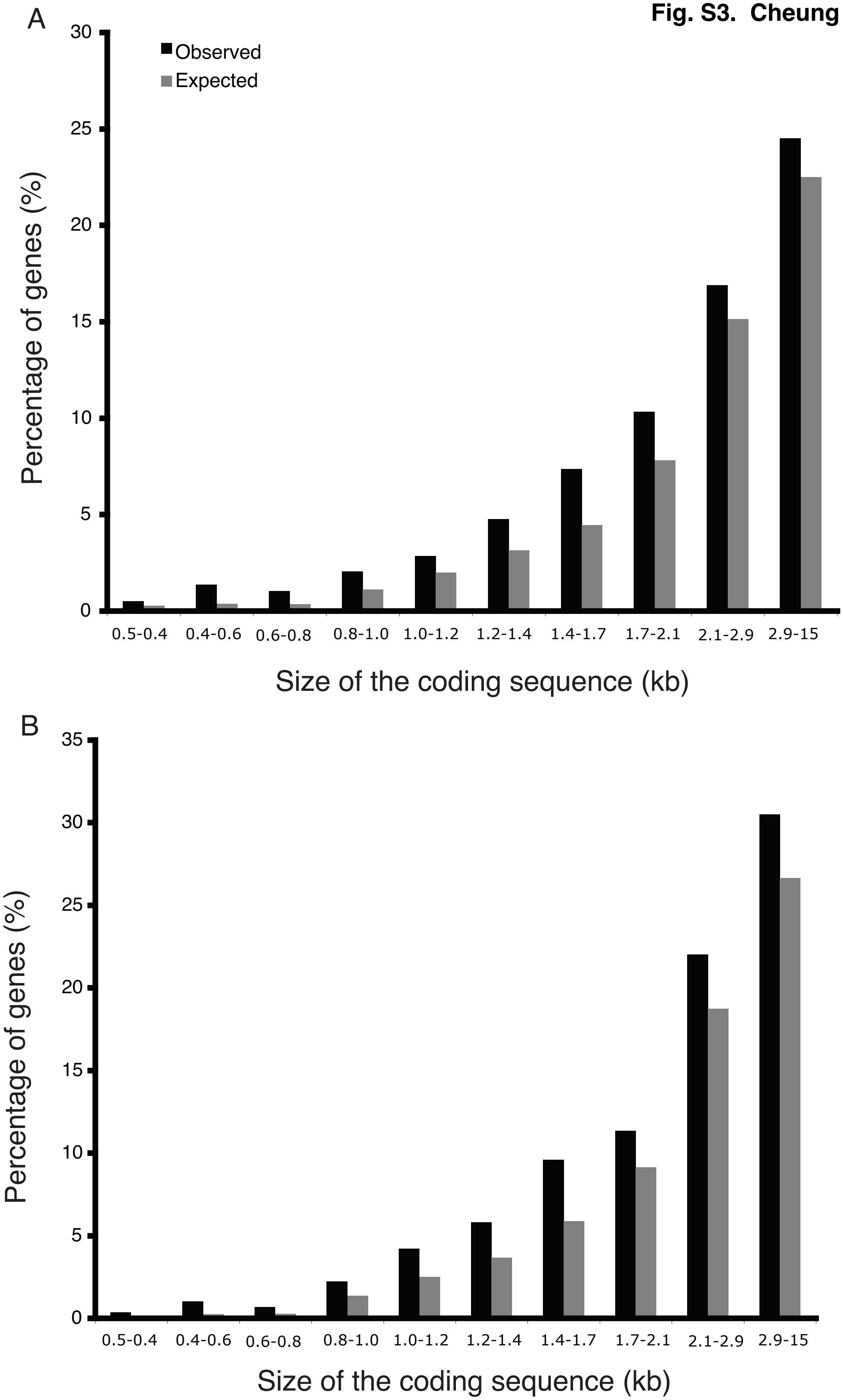

Supplement: Supplementary file 3 [file pbio.0060277.sg003.pdf]
