# Supplementary material for: Chromatin- and Transcription-Related Factors Repress Transcription from within Coding Regions throughout the Saccharomyces cerevisiae Genome
Source: PLoS Biol. 2008 Nov 11;6(11):e277. doi: 10.1371/journal.pbio.0060277 (PMC2581627; doi:10.1371/journal.pbio.0060277)
Supplement: Supplementary file 6 [file pbio.0060277.st001.doc]

Table S1. Histone H3 mutants that allow cryptic initiation

| mutation | amino acid change | DNA bp change | viability as only H3 gene |
| --- | --- | --- | --- |
| *HHT1-105* | E105K | G316A | - |
| *HHT1-130* | L130Y | T392A | -/+ |
| *HHT2-151* | 151N | T155A | -/+ |
| *HHT2-152* | 151S | T155G | - |
| *HHT2-55* | Q55H | A168T | - |
| *HHT2-57* | S57P | T172C | - |
| *HHT2-106* | E105K | G316A |  |
| *HHT2-118* | T118F | A335T, C336T | -/+ |
