# Supplementary material for: Chromatin- and Transcription-Related Factors Repress Transcription from within Coding Regions throughout the Saccharomyces cerevisiae Genome
Source: PLoS Biol. 2008 Nov 11;6(11):e277. doi: 10.1371/journal.pbio.0060277 (PMC2581627; doi:10.1371/journal.pbio.0060277)
Supplement: Supplementary file 7 [file pbio.0060277.st005.doc]

Table S5. TATA consensus sequences in coding regions

|  |  | Is there a TATA consensus sequence in the coding region? | |
| --- | --- | --- | --- |
| Mutant | Does the gene have a cryptic promoter? | Yes | No |
| *spt6-1004* | yes | 422 | 538 |
| *spt6-1004* | no | 1006 | 3908 |
|  |  |  |  |
| *spt16-197* | yes | 516 | 614 |
| *spt16-197* | no | 912 | 3827 |
