# Supplementary material for: Chromatin- and Transcription-Related Factors Repress Transcription from within Coding Regions throughout the Saccharomyces cerevisiae Genome
Source: PLoS Biol. 2008 Nov 11;6(11):e277. doi: 10.1371/journal.pbio.0060277 (PMC2581627; doi:10.1371/journal.pbio.0060277)
Supplement: Supplementary file 8 [file pbio.0060277.st007.doc]

**Table S7.** Genes identified that express short proteins in an *spt6-1004* mutant

predicted	estimated

gene	short transcript	estimated	short protein

gene	length	length^a^	protein size^b^	size^b^

*AMS1*	3252 bp	698 – 1336 bp	120 kD	45 kD

*APM2*	1818 bp	411 – 763 bp	78 kD	18 kD

*CCR4*	2514 bp	550 – 1041 bp	93 kD	25 kD

*CHS6*	2241 bp	932 – 1368 bp	85 kD	42 kD

*DBF20*	1695 bp	713 – 1040 bp	63 kD	32 kD

*DDC1*	1839 bp	415 – 771 bp	70 kD	15 kD

*MSH1*	2880 bp	1751 – 2315 bp	94 kD	65 kD

*MUS81*	1899 bp	795 – 1163 bp	73 kD	21 kD

*OMS1*	1416 bp	1144 – 1416 bp	52 kD	42 kD

*PET56*	1239 bp	1003 – 1239 bp	46 kD	40 kD

*PSK2*	3306 bp	1358 – 2007 bp	120 kD	61 kD

*PUS4*	1212 bp	751 – 981 bp	54 kD	37 kD

*RUP1*	2016 bp	1233 – 1624 bp	85 kD	65 kD

*SGF73*	1975 bp	443 – 726 bp	75 kD	22 kD

*SOV1*	2698 bp	1643 – 2170 bp	95 kD	67 kD

*SPB4*	1821 bp	1 - 412 bp	65 kD	10 kD

*STE11*	2154 bp	1316 – 1735 bp	85 kD	45 kD

*SYF1*	2580 bp	563 – 1067 bp	95 kD	34 kD

*VPS8*	3825 bp	1565 - 2318 bp	110 kD	30 kD

*YNL234W*	1281 bp	1036 – 1281 bp	45 kD	33 kD

^a^ Based on *spt6-1004* microarray results

^b^ Based on α-TAP Western analysis
