# Supplementary material for: Chromatin- and Transcription-Related Factors Repress Transcription from within Coding Regions throughout the Saccharomyces cerevisiae Genome
Source: PLoS Biol. 2008 Nov 11;6(11):e277. doi: 10.1371/journal.pbio.0060277 (PMC2581627; doi:10.1371/journal.pbio.0060277)
Supplement: Supplementary file 9 [file pbio.0060277.st009.doc]

**Table S10.** *Saccharomyces cerevisiae* strains used in this study

Name	Genotype

FY4	*MAT***a**	(prototroph)

FY70	*MAT*α	*leu2Δ1*

FY80	*MAT***a**	*leu2Δ1 lys2-128δ trp1Δ63 ura3-52*

FY103	*MAT***a**	*leu2Δ1 lys2-128δ trp1Δ63 ura3-52 spt10-302::URA3*

FY244	*MAT*α	*ura3-52 spt4Δ0::URA3*

FY347	*MAT*α	*leu2Δ1 spt16-197*

FY390	*MAT***a**	*leu2Δ1 lys2-128Δ ura3-52 (hta1-htb1) Δ0::LEU2*

FY1725	*MAT***a**	*his3Δ200 ura3-52 chd1Δ0::HIS3*

FY2283	*MAT*α	*his3Δ200 leu2Δ1 lys2-128Δ ura3Δ0 hir1Δ201::kanMX*

FY2425	*MAT*α	*his3Δ200 leu2Δ1 lys2-128Δ ura3-52 spt6-1004-FLAG*

FY2445	*MAT***a**	*his3Δ200 lys2-128Δ ura3-52 kanMX-GAL1pr-FLO8-HIS3*

*RPB3-HA1::LEU2 spt2Δ0::kanMX SPT6-FLAG*

FY2482	*MAT*α	*his3Δ200 leu2Δ0 lys2-128Δ ura3Δ0 spt21Δ0::kanMX*

FY2712	*MAT***a**	*his3Δ200 leu2Δ1 lys2-128Δ trp1Δ63 ura3-52 kanMX-GAL1pr-FLO8-HIS3*

FY2713	*MAT*α	*his3Δ200 leu2Δ1 lys2-128Δ trp1Δ63 ura3-52 kanMX-GAL1pr-FLO8-HIS3*

FY2714	*MAT*α	*arg4-12 his3Δ200 lys2-128Δ ura3-52 kanMX-GAL1pr-FLO8-HIS3*

FY2715	*MAT***a**	*arg4-12 his3Δ200 lys2-128Δ ura3-52 kanMX-GAL1pr-FLO8-HIS3*

FY2716	*MAT*α	*his3Δ200 leu2Δ1 lys2-128Δ ura3-52 kanMX-GAL1pr-FLO8-HIS3*

FY2717	*MAT***a**	*arg4-12 his3Δ200 lys2-128Δ ura3-52 kanMX-GAL1pr-FLO8-HIS3*

*[SPT10::URA3]dup*

FY2718	*MAT*α	*his3Δ200 leu2Δ1 lys2-128Δ ura3-52 kanMX-GAL1pr-FLO8-HIS3*

*[SPT10::URA3]dup*

FY2719	*MAT*α	*his3Δ200 leu2Δ1 lys2-128Δ trp1Δ63 ura3-52 kanMX-GAL1pr-FLO8-HIS3*

*spt6-1004-FLAG*

FY2720	*MAT***a**	*arg4-12 his3Δ200 lys2-128Δ ura3-52 kanMX-GAL1pr-FLO8-HIS3*

*spt6-1004-FLAG*

FY2721	*MAT***a**	*arg4-12 his3Δ200 lys2-128Δ ura3-52 kanMX-GAL1pr-FLO8-HIS3*

*spt16-197*

FY2722	*MAT***a**	*arg4-12 his3Δ200 leu2Δ1 lys2-128Δ trp1Δ63 ura3-52*

*kanMX-GAL1pr-FLO8-HIS (hta1-htb1) Δ0::LEU2*

FY2723	*MAT*α	*his3Δ200 leu2Δ1 lys2-128Δ ura3-52 kanMX-GAL1pr-FLO8-HIS3*

*hht2-57*

FY2724	*MAT*α	*his3Δ200 leu2Δ1 lys2-128Δ trp1Δ63 ura3-52 kanMX-GAL1pr-FLO8-HIS3*

*hht2-105*

FY2725	*MAT***a**	*arg4-12 his3Δ200 lys2-128Δ ura3-52 kanMX-GAL1pr-FLO8-HIS3*

*ctk1Δ0::URA3*

FY2726	*MAT***a**	*arg4-12 his3Δ200 lys2-128Δ ura3-52 kanMX-GAL1pr-FLO8-HIS3*

*ctk2Δ0::URA3*

FY2727	*MAT***a**	*arg4-12 his3Δ200 lys2-128Δ ura3-52 kanMX-GAL1pr-FLO8-HIS3*

*spt21Δ0::kanMX*

FY2728	*MAT***a**	*arg4-12 his3Δ200 leu2Δ1 lys2-128Δ ura3-52 kanMX-GAL1pr-FLO8-HIS3*

*hir1Δ0::LEU2*

FY2729	*MAT***a**	*ura3Δ0 ctk1Δ0::URA3*

FY2730	*MAT***a**	*ura3Δ0 ctk2Δ0::URA3*

FY2731	*MAT***a**	*set2Δ0::kanMX*

FY2732	*MAT***a**	*ras2Δ0::kanMX*

FY2733	*MAT***a**	*RAS2^val19^*

L1102	*MAT*α	*his3Δ200 leu2Δ1 lys2Δ0 can1Δ0::STE2pr-LEU2 FLO8-HIS3*

L1103	*MAT***a**	*his3Δ200 leu2Δ0 ura3Δ0 kanMX-GAL1pr-FLO8-HIS3 ctk3Δ0::kanMX*

L1104	*MAT***a**	*arg4-12 his3Δ200 lys2-128Δ ura3-52 kanMX-GAL1pr-FLO8-HIS3*

*set2Δ0::kanMX*

L1105	*MAT***a**	*arg4-12 his3Δ200 leu2Δ0 ura3Δ0 kanMX-GAL1pr-FLO8-HIS3*

*eaf3Δ0::kanMX*

L1106	*MAT***a**	*his3Δ200 ura3Δ0 kanMX-GAL1pr-FLO8-HIS3 rco1Δ0::kanMX*

L1107	*MAT*α	*his3Δ200 leu2Δ0 ura3-52 kanMX-GAL1pr-FLO8-HIS3 asf1Δ0::kanMX*

L1108	*MAT***a**	*his3Δ200 leu2Δ0 ura3-52 kanMX-GAL1pr-FLO8-HIS3 rtt106Δ0::kanMX*

L1109	*MAT*α	*his3Δ1 lys2-128Δ met15Δ0 ura3Δ0 kanMX-GAL1pr-FLO8-HIS3*

*chd1Δ0::kanMX*

L1110	*MAT***a**	*arg4-12 his3Δ1 leu2Δ0 lys2-128Δ ura3Δ0 kanMX-GAL1pr-FLO8-HIS3*

*rtf1Δ0::kanMX*

L1111	*MAT*α	*arg4-12 his3Δ200 ura3-52 kanMX-GAL1pr-FLO8-HIS3 cdc73Δ0::kanMX*

L1112	*MAT***a**	*his3Δ200 leu2Δ1 lys2-128Δ ura3Δ0 can1Δ0::STE2pr-LEU2*

*APM2-TAP::His3MX spt6-1004-FLAG ygr117cΔ0::kanMX*

L1113	*MAT***a**	*his3Δ1 leu2Δ0 can1Δ0::STE2pr-LEU2 DDC1-TAP::His3MX*

*spt6-1004-FLAG ygr117cΔ0::kanMX*

L1114	*MAT***a**	*his3Δ200 leu2Δ1 lys2-128Δ can1Δ0::STE2pr-LEU2 OMS1-TAP::His3MX*

*spt6-1004-FLAG ygr117cΔ0::kanMX*

L1115	*MAT***a**	*his3Δ1 leu2Δ0 lys2-128Δ ura3Δ0 can1Δ0::STE2pr-LEU2*

*PUS4-TAP::His3MX spt6-1004-FLAG ygr117cΔ0::kanMX*

L1116	*MAT***a**	*his3Δ200 leu2Δ0 lys2-128Δ  met15Δ0 can1Δ0::STE2pr-LEU2*

*SYF1-TAP::His3MX spt6-1004-FLAG ygr117cΔ0::kanMX*

L1117	*MAT***a**	*his3Δ1 leu2Δ0 met15Δ0 ura3Δ0 apm2-1-TAP::His3MX*

L1118	*MAT***a**	*his3Δ1 leu2Δ0 met15Δ0 ura3Δ0 apm2-2-TAP::His3MX*

L1119	*MAT***a**	*his3Δ1 leu2Δ0 met15Δ0 ura3Δ0 apm2-3-TAP::His3MX*

L1120	*MAT***a**	*his3Δ1 leu2Δ0 ura3-52 spt6-1004-FLAG apm2-1-TAP::His3MX*

L1121	*MAT***a**	*his3Δ200 leu2Δ1 met15Δ0 ura3Δ0 spt6-1004-FLAG*

*apm2-2-TAP::His3MX*

L1122	*MAT***a**	*his3Δ1 leu2Δ0 ura3-52 spt6-1004-FLAG apm2-3-TAP::His3MX*

L1123	*MAT***a**	*his3Δ1 leu2Δ0 met15Δ0 ura3Δ0 pus4-1-TAP::His3MX*

L1124	*MAT***a**	*his3Δ1 leu2Δ0 met15Δ0 ura3Δ0 pus4-2-TAP::His3MX*

L1125	*MAT***a**	*his3Δ1 leu2Δ0 met15Δ0 ura3Δ0 pus4-3-TAP::His3MX*

L1126	*MAT***a**	*his3Δ1 leu2Δ0 lys2-128Δ ura3-52 spt6-1004-FLAG pus4-1-TAP::His3MX*

L1127	*MAT*α	*his3Δ200 leu2Δ1 ura3Δ0 spt6-1004-FLAG pus4-2-TAP::His3MX*

L1128	*MAT***a**	*his3Δ1 leu2Δ0 lys2-128Δ ura3-52 spt6-1004-FLAG pus4-3-TAP::His3MX*
